# Identification of Critical Genes for Ovine Horn Development Based on Transcriptome during the Embryonic Period

**DOI:** 10.3390/biology12040591

**Published:** 2023-04-13

**Authors:** Yuanyuan Luan, Shangjie Wu, Mingkun Wang, Yabin Pu, Qianjun Zhao, Yuehui Ma, Lin Jiang, Xiaohong He

**Affiliations:** 1 Institute of Animal Sciences, Chinese Academy of Agricultural Sciences (CAAS), Beijing 100193, China; 82101205302@caas.cn (Y.L.); 82101215392@caas.cn (S.W.); mkWang2000@163.com (M.W.); puyabin@caas.cn (Y.P.); zhaoqianjun@caas.cn (Q.Z.); mayuehui@caas.cn (Y.M.); jianglin@caas.cn (L.J.); 2 Key Laboratory of Livestock and Poultry Resources Evaluation and Utilization, Institute of Animal Sciences, Chinese Academy of Agricultural Sciences (CAAS), Beijing 100193, China

**Keywords:** fetal sheep, RNA-seq, horn development, horn bud, *RXFP2*

## Abstract

**Simple Summary:**

A unique structure of ruminants, the horn trait is not only closely related to natural and sexual selection but is also an important trait for polled sheep breeding. *RXFP2* may be a crucial gene in regulating sheep horn. However, the underlying genetic mechanisms of sheep horn development remain largely unknown. In this study, we investigated the gene expression profile of the horn buds in sheep fetuses using RNA-seq technology. We identified 68 differentially expressed genes in the horn buds of 105-day-old Altay sheep fetuses, including *RXFP2*, *FOXL2*, and *TNN*. Further, we found that the Wnt signaling pathway may be responsible for horn development. Our study provides new possible marker genes for horn development, which may promote our understanding of the genetic mechanisms of sheep horn formation.

**Abstract:**

Horns, also known as headgear, are a unique structure of ruminants. As ruminants are globally distributed, the study of horn formation is critical not only for increasing our understanding of natural and sexual selection but also for the breeding of polled sheep breeds to facilitate modern sheep farming. Despite this, a significant number of the underlying genetic pathways in sheep horn remain unclear. In this study, to clarify the gene expression profile of horn buds and investigate the key genes in horn bud formation, RNA-sequencing (RNA-seq) technology was utilized to investigate differential gene expression in the horn buds and adjacent forehead skin of Altay sheep fetuses. There were only 68 differentially expressed genes (DEGs) identified, consisting of 58 up-regulated genes and 10 down-regulated genes. *RXFP2* was differentially up-regulated in the horn buds and had the highest significance (*p*-value = 7.42 × 10^−14^). In addition, 32 DEGs were horn-related genes identified in previous studies, such as *RXFP2*, *FOXL2*, *SFRP4*, *SFRP2*, *KRT1*, *KRT10*, *WNT7B*, and *WNT3*. Further, Gene Ontology (GO) analysis showed that the DEGs were mainly enriched with regard to growth, development, and cell differentiation. Pathway analysis revealed that the Wnt signaling pathway may be responsible for horn development. Further, through combining the protein–protein interaction networks of the DEGs, it was found that the top five hub genes, namely, *ACAN*, *SFRP2*, *SFRP4*, *WNT3*, and *WNT7B*, were also associated with horn development. Our results suggest that only a few key genes, including *RXFP2*, are involved in bud formation. This study not only validates the expression of candidate genes identified at the transcriptome level in previous studies but also provides new possible marker genes for horn development, which may promote our understanding of the genetic mechanisms of horn formation.

## 1. Introduction

Horns, also termed headgear, are a unique structure developed by ruminants. Sheep (Ovis aries) is an important domesticated ruminant that is widely distributed around the world and that has provided milk, skin, meat, and wool for humans [1]. Sheep horns are hollow paired structures with a skeletal core that is covered by the integument; located on frontal bones, they may develop from neural crest stem cells [2,3]. Able to be used as a weapon, horns play a significant role in defending against predators and in sexual selection through their use in intra-male competition [4,5,6,7,8,9]. The study of horn formation and the presence or absence of horns is critical not only for the study of natural and sexual selection but also for the breeding of polled sheep breeds to facilitate modern sheep farming.

The genetic mapping of the polled phenotype in sheep has been investigated in previous studies. The sheep polled locus has been found to be located on chromosome 10 in a hybrid population of Merino and Romney sheep [10] and Soay sheep [11]. Genome-wide signal selection analysis found a significant SNP locus (OAR10_29546872) on chromosome 10 in the polled phenotype, which is adjacent to the relaxin family peptide receptor 2 (*RXFP2*) gene [12]. Further, a study of Soay sheep found that an SNP in the 3′-UTR region of the *RXFP2* gene was highly correlated with the polled trait [8]. Meanwhile, Wiedemar et al. [13] found that a 1.8 kb insertion in the 3′-UTR region of the *RXFP2* gene was associated with the polled phenotype in five European sheep breeds. Conversely, an analysis of 34 European sheep breeds and three Chinese sheep breeds reported that an insertion in the 3′-UTR region of the *RXFP2* gene was not associated with the polled phenotype in some breeds [14,15], a finding that suggests that the sheep horn is more genetically complex than initially thought. In addition, previous studies have found that a series of genes and proteins, such as *RXFP2* and tenascin N (*TNN*), are related to horn length, growth direction, and deformity [7,16,17]. Lately, comparative transcriptome analysis has demonstrated that bovine horns and cervid antlers most likely share the same cellular origin, namely from neural crest stem cells [18]. Despite this, a significant number of the underlying genetic pathways in sheep horn remain unclear.

The Altay sheep is an ancient indigenous breed that is distributed in Xinjiang Province of China. All Altay rams have two horns, while some ewes have two horns and others are hornless; this breed, therefore, provides an ideal model for the study of the genetic basis of sheep horns. Previous studies have demonstrated that sheep horn formation is initiated during the embryonic period [3,19]. However, our understanding of horn bud formation in sheep is very limited. It is not known whether the genes identified in previous studies are critical to horn development or which key genes and pathways are involved in the formation of horns. RNA sequencing (RNA-seq) is a high-throughput technology that provides a comprehensive view of the entire transcriptome, which contributes to a better understanding of embryonic development and the role of genes [20,21].

In this study, transcriptome analysis was performed on the horn buds and adjacent forehead skin of sheep fetuses to clarify the gene expression profile of horn buds and investigate whether the horn candidate genes identified in previous studies affect horn bud formation. *RXFP2*, the most important candidate gene screened at the genome level, was found to be differentially up-regulated in the horn bud tissues and recorded the highest significance (*p*-value = 7.42 × 10^−14^). In addition, a series of key genes for horn development were identified and the Wnt pathway was found to potentially be responsible for horn development. The results of this study may promote our understanding of the genetic mechanisms of horn formation.

## 2. Materials and Methods

### 2.1. Sample Collection

According to our previous histological results (unpublished), horn buds initially grow from the epithelium and undergo rapid development during the embryonic period of ~100 days in Altay sheep. Therefore, to exclude the influence of gender on the horn phenotype [7,8], three 105-day-old male sheep fetuses were used in this study. During tissue collection, it was observed that the hairs on the head of sheep fetuses were spirally shaped in horn bud regions. When the hair was removed, an indentation in the skin was found that was not present in the adjacent parts of the skin. The shape of sheep horn buds is similar to that of cattle horn buds [22]. Horn buds (H) and forehead skin tissues (S) adjacent to the horn buds from the same individual were collected. The samples were placed in RNAlater and stored at −80 °C in a refrigerator for further analysis.

### 2.2. Preparation of Histological Samples

The horn buds and skin tissues were fixed in a 4% paraformaldehyde solution, dehydrated in a graded ethanol series, cleared with xylene, and embedded in paraffin. Hematoxylin–eosin (HE) was used to stain the microtome slices. A slice digital scanner (Pannoramic 250FLASH, Budapest, Hungary) was utilized to scan and image the entirety of the slices. Images of HE slices at 10× magnification were captured using the software SlideViewer, and epithelial thickness was calculated using Image J. At least 10 parts were selected from each slice randomly to detect the epithelial thickness. All data are presented as the means ± standard deviation (SD).

### 2.3. RNA Extraction, cDNA Library Preparation, and Sequencing

We used Trizol Reagent (Invitrogen, Carlsbad, CA, USA) to extract total RNA and used an Agilent 2100 bioanalyzer (Agilent, Santa Clara, CA, USA) to measure RNA concentration and integrity. For the sequencing analysis, samples with RNA concentration >100 ng/μL, total RNA > 2 μg, and RIN > 7.0 were chosen. Six libraries were constructed, with 2 μg of RNA per sample. A VAHTS mRNA-seq v2 Library Prep Kit for Illumina was used to cluster the index-coded samples. According to manufacturer instructions, the libraries were sequenced on an Illumina NovaSeq6000 RNA-Seq platform to produce 150 bp paired-end reads.

### 2.4. Quality Control and Mapping of Reads

Primary quality control of raw data (raw reads) was carried out in fastq format. Raw data (raw reads) with adaptor reads and low-quality reads were removed using quality control standards developed by Berry Genomics Beijing Co., Ltd., Beijing, China. These standards were as follows: clean data (clean reads) were obtained by removing read pairs that contain N more than 3 or theproportion of base with a quality value below 5 is more than 20%, in any end, or adapter sequence was founded. HISAT2 (v2.1.0) software was used to align clean reads with the sheep reference genome (Oar rambouillet v1.0).

### 2.5. Analysis of Differentially Expressed Genes

The featureCounts program in Subread (v2.0.3) was used to calculate the gene expression of horn buds and skin tissues [23]. Moreover, the expression of a gene was expressed by FPKM value. FPKM, which stands for fragments per kilobase of exon per million mapped fragments, was transferred from read counts. The package DESeq2 v1.36.0 of R software [24] was used to examine DEGs in horn buds and skin tissues. The DEGs were selected using the thresholds of *p*-value < 0.05 and |log2FoldChange| > 1. The heatmaps included in the R v4.2.2 software package were subsequently utilized to perform a clustering analysis of the detected DEGs.

### 2.6. Enrichment Analysis

To further understand the functions of DEGs in horn buds and forehead skin tissues in sheep, Gene Ontology (GO) and the Kyoto Encyclopedia of Genes and Genomes (KEGG) were used for enrichment analysis. This was carried out on the g: Profiler (https://biit.cs.ut.ee/gprofiler/gost (accessed on 15 September 2022)) and KEGG Mapper [25,26] (https://www.genome.jp/kegg/mapper/color.html (accessed on 15 September 2022)) tools. All analyses were deemed substantially enriched if the *p*-value was less than 0.05. All *p* values were adjusted using Benjamini and Hochberg’s FDR correction method.

### 2.7. Protein-Protein Interaction Network Construction

We used the STRING database (https://string-db.org (accessed on 22 September 2022) version 11.5) to assess the interactions of all DEGs in horn buds and forehead skin tissues. We used the Ovis aries genome as a reference, and the confidence value was set at 0.4. PPI networks are represented as graphs in Cytoscape, with nodes representing proteins and edges representing related interactions. The hub genes were identified with the plug-in cytoHubba of Cytoscape.

### 2.8. Quantitative Real-Time PCR (qRT-PCR) for Verification of DEGs

Six genes were chosen at random for qRT-PCR to assess transcriptome sequencing accuracy. Using Primer-BLAST (https://www.ncbi.nlm.nih.gov/tools/primer-blast/ (accessed on 5 October 2022)) to create primers (see Supplementary Table S1). RNA was generated into single-stranded cDNA using a HiScript III All-in-one RT SuperMix Ideal (Vazyme, Nanjing, China) for qPCR. The synthesized cDNA was used as the template for qRT-PCR using Taq Pro Universal SYBR qPCR Master Mix (Vazyme, Nanjing, China) by the ABI 7900 real-time PCR instrument (Applied Biosystems). The six genes were tested in triplicate using the six samples. The DEGs’ expression was compared to PGK1, an internal control [27], utilizing Livak and Schmittgen’s 2^−∆∆CT^ technique to derive quantification cycle values [28].

## 3. Results

### 3.1. Histological Appearance

**Figure 1:**
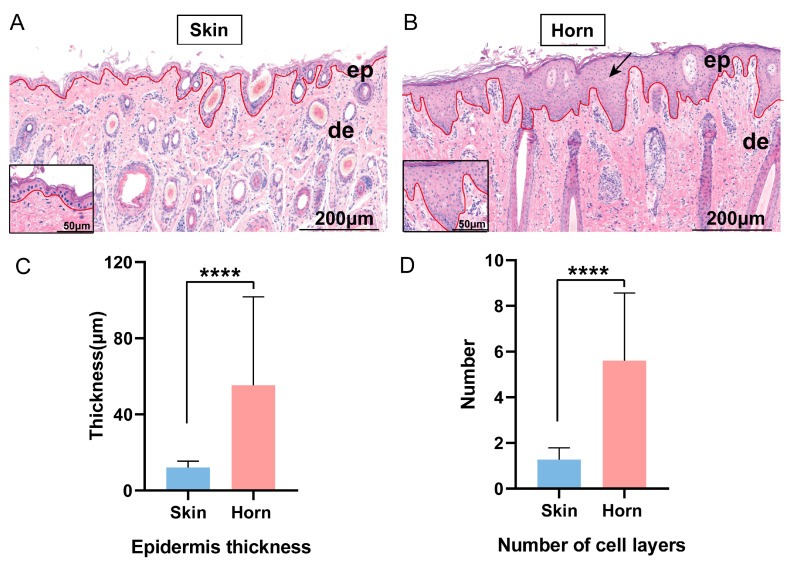
Histological appearance of samples. (**A**) Forehead skin with one or two epithelial layers. (**B**) Horn buds with multiple epithelial layers. The red line marks the epidermis and the black arrow marks the epidermis of the horn buds. The insets show changes in the number of epithelial cell layers in the horn buds and skin tissues. (**C**) Epidermis thickness of horn buds and skin tissues. (**D**) The number of epithelial cell layers in the horn bud region. **** *p* < 0.0001. ep, epidermis; de, dermis.

To analyze the morphological features of horn bud development, the histological changes in the embryonic horn buds were evaluated using hematoxylin–eosin (HE). The horn buds of the 105-day-old male sheep fetuses showed an obvious difference in the epithelial portion compared with the forehead skin. The histological results showed that the horn bud epithelium was thicker than the skin epithelium in fetal sheep (Figure 1A–C). Further, the number of epithelial cell layers in the horn bud region was significantly higher than that in the forehead skin tissue (Figure 1A,B,D).

### 3.2. Overview of Transcriptomics Data

**Figure 2:**
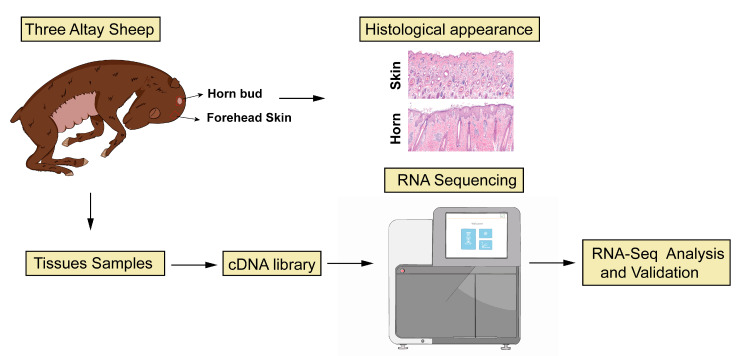
Schematic diagram of technical route.

**Figure 3:**
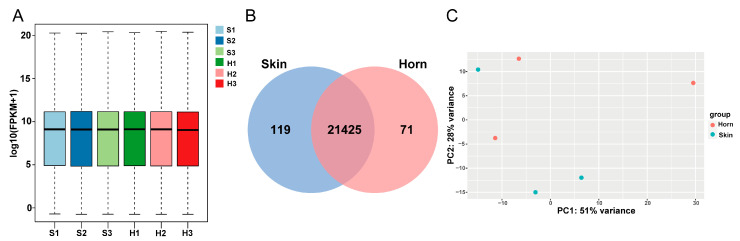
Characteristics of RNA-seq data. (**A**) Distribution of FPKM in a box plot across six samples. (**B**) Venn diagram of genes expressed in horn buds and skin tissues. (**C**) The PCA analysis. S, forehead skin; H, horn bud.

In order to determine the differences in gene expression between the horn buds and skin tissues of the sheep fetuses, RNA-seq was used to analyze the transcript expression profiles (Figure 2). A total of 247.46 million raw reads were obtained by RNA-seq for six samples from the S and H groups. An average of 39.69 million clean reads were generated after quality control was carried out. About 94.97% of the clean reads on average were successfully mapped to the Ovis aries reference genome (95.37% for the S group; 94.57% for the H group) (Table 1). The FPKM distribution box plot showed that the S and H groups had similar gene expression distributions (Figure 3A). Of these, 71 genes were specifically expressed in the horn buds and 119 genes were specifically expressed in the skin tissues, and 21,425 genes were expressed in both tissues (Figure 3B). PCA analysis showed that the samples were not separated by different groups (H and S groups) (Figure 3C). These results indicated that the gene expression profiles of the horn bud region and its adjacent forehead skin were similar in the global transcriptome, suggesting that horn development may be regulated by a few key genes during embryonic development.

### 3.3. Differentially Expressed Genes Analysis

**Figure 4:**
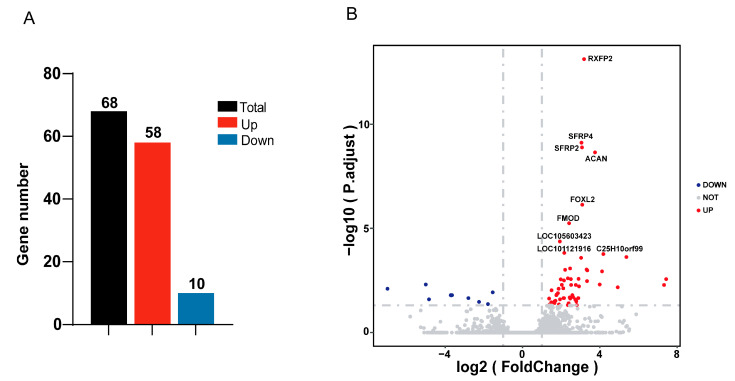
Identification of DEGs. (**A**) The statistical analysis of DEGs in H (horn bud) and S (forehead skin) groups. (**B**) Volcano plot showing DEGs (*p* value < 0.05) in H (horn bud) and S (forehead skin) groups.

**Figure 5:**
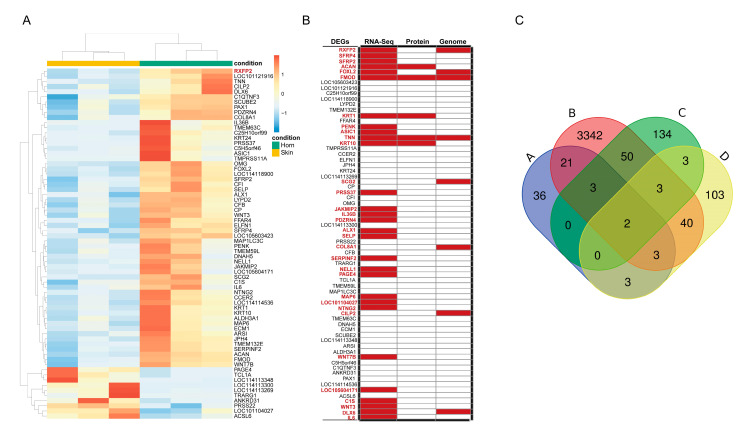
Analysis of DEGs. (**A**) The clustering analysis of the DEGs is shown on a heatmap. Different genes are represented by rows and samples are represented by columns. (**B**) Overlapping DEGs from this study and horn-related genes from previous studies. Red represents the overlapping genes. (**C**) Venn diagram of DEGs and horn-related genes. A: DEGs; B: Horn-associated genes in transcriptome studies by Wang, Y. et al. [3], Pannetier, M. et al. [33], and Boulanger, L. et al. [34]; C: Horn-associated genes in proteome studies by He, X. et al. [17]; D: Horn-associated genes in genome studies by Li, X. et al. [1], Montgomery, G.W. et al. [10], Kijas, J.W. et al. [12,31], Wiedemar, N. et al. [13], Lühken, G. et al. [14], He, X. et al. [15], Wang, X. et al. [29], Ren, X. et al. [30], Pailhoux, E. et al. [32], and Medugorac, I. et al. [35].

Based on RNA-seq data, 68 DEGs were identified (|log2FoldChange| > 1, *p*-value < 0.05), consisting of 58 up-regulated genes and 10 down-regulated genes (Figure 4A,B). Supplementary Table S2 provides information on the DEGs. The DEGs were further distinguished by supervised hierarchical clustering (Figure 4A). Interestingly, we found that *RXFP2*, an important candidate gene for the sheep horn trait, was highly expressed in the horn buds, recording the highest significance (*p*-value = 7.42 × 10^−14^). Meanwhile, we integrated the gene sets (Table S3) and compared them with the DEGs identified in this study; these sets included the horn-related genes and proteins of sheep, goats, and cattle horns identified in previous studies [1,3,10,12,13,14,15,17,29,30,31,32,33,34,35]. We found that 32 of the DEGs were horn-related genes and proteins identified in these previous studies, accounting for 47% of all the DEGs (Figure 5B,C); these DEGs included *RXFP2*, forkhead box L2 (*FOXL2*), secreted frizzled-related protein 4 (*SFRP4*), secreted frizzled-related protein 2 (*SFRP2*), keratin 1 (*KRT1*), keratin 10 (*KRT10*), Wnt family member 7B (*WNT7B*), and Wnt family member 3 (*WNT3*) (Figure 5B,C). Further, among the DEGs, tenascin N (*TNN*) and fibromodulin (*FMOD*) were identified as horn-related genes in previous studies on three levels, namely the genome, transcriptome, and proteome levels.

### 3.4. Analysis of Gene Function Enrichment

**Figure 6:**
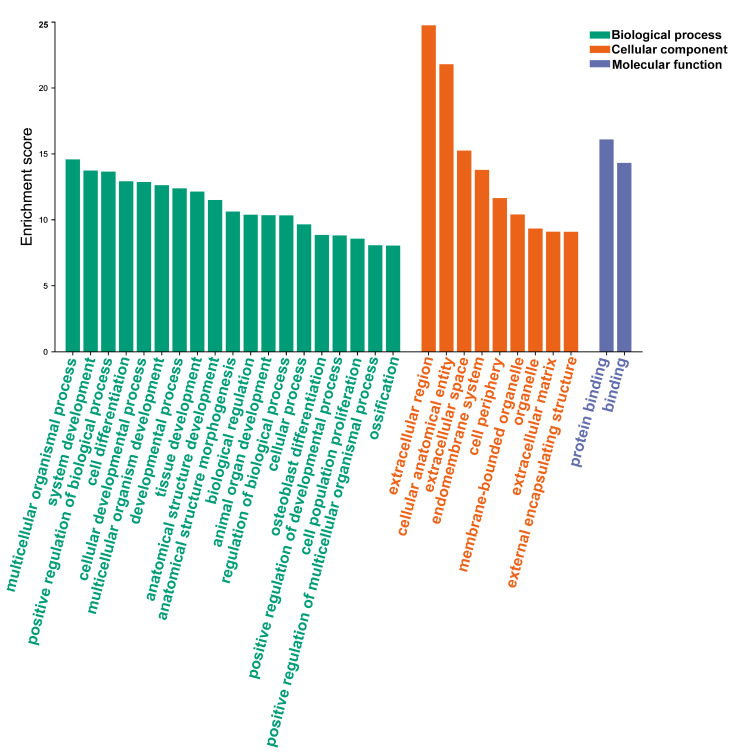
Top 30 enriched GO terms of DEGs in horn buds and forehead skin tissues. The x-axis displays the GO terms, while the y-axis displays the number of DEGs.

To further investigate the function of the DEGs in the skin and horn buds, GO analysis of the DEGs was carried out using the g: Profiler. A total of 56 DEGs were enriched to GO terms (*p* < 0.05), and 134 biological process terms, 41 cellular component terms, and 15 molecular function terms were significantly enriched. The results are shown in Supplementary Table S4. The top 30 GO terms were identified including growth and development terms, such as the cellular developmental process, system development, tissue development, and animal organ development terms (Figure 6). Interestingly, some DEGs were found to be mainly involved in osteoblast differentiation, ossification, and cell differentiation.

**Figure 7:**
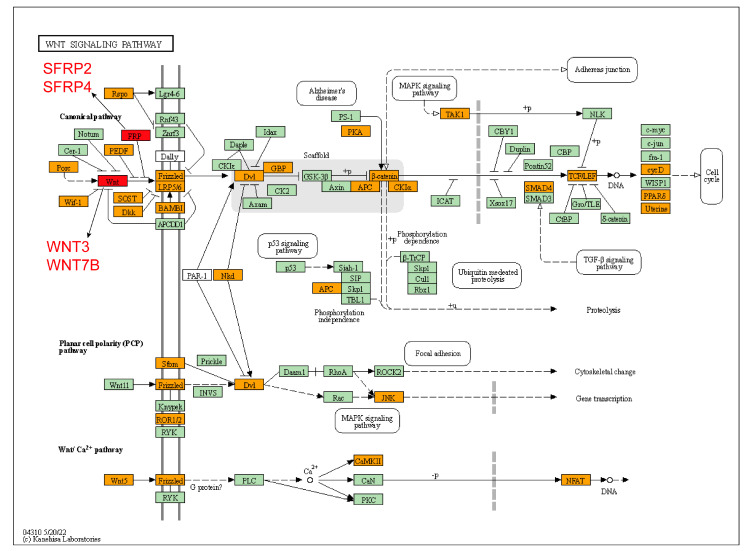
KEGG pathway map for Wnt signaling pathway. The genes form our study are shown in red and those from previous studies are shown in yellow.

The KEGG enrichment analysis showed that the DEGs were mainly enriched in the Wnt signaling pathway (Figure S1) which included the *SFRP4*, *SFRP2*, *WNT7B*, and *WNT3* genes. We performed the KEGG analysis by merging the genes from two different gene sets and the DEGs from this study and the horn-related gene sets from previous studies. Interestingly, the KEGG enrichment analysis showed that these DEGs were also mainly enriched in the Wnt signaling pathway (Figure 7), which suggests that the Wnt signaling pathway may play a significant role in horn development.

### 3.5. Protein–Protein Interaction Networks of the DEGs

**Figure 8:**
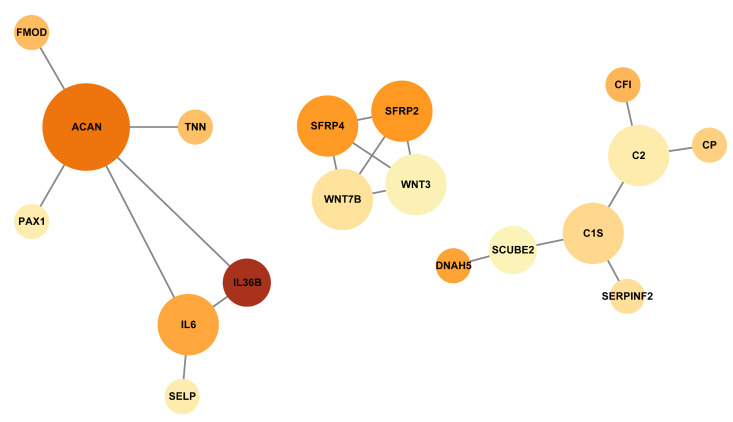
Protein–protein interaction networks of the DEGs. The circle size represents the degree and the circle color represents the log2FoldChange value. The size of the circles ranges from 1 to 6, and the number of colors ranges from 1.4 to 5.0.

We used the STRING database to investigate the relationships between the DEGs in order to identify the hub genes involved in the process of horn development. The protein–protein interaction networks (PPI) of the DEGs were found to have 18 nodes and 19 edges. (Figure 8). The top five hub genes, which were identified using the plugin cytoHubba of Cytoscape, were found to be aggrecan (*ACAN*), *SFRP2*, *SFRP4*, *WNT3*, and *WNT7B* (Table S5).

### 3.6. Validation of RNA-Seq Data

**Figure 9:**
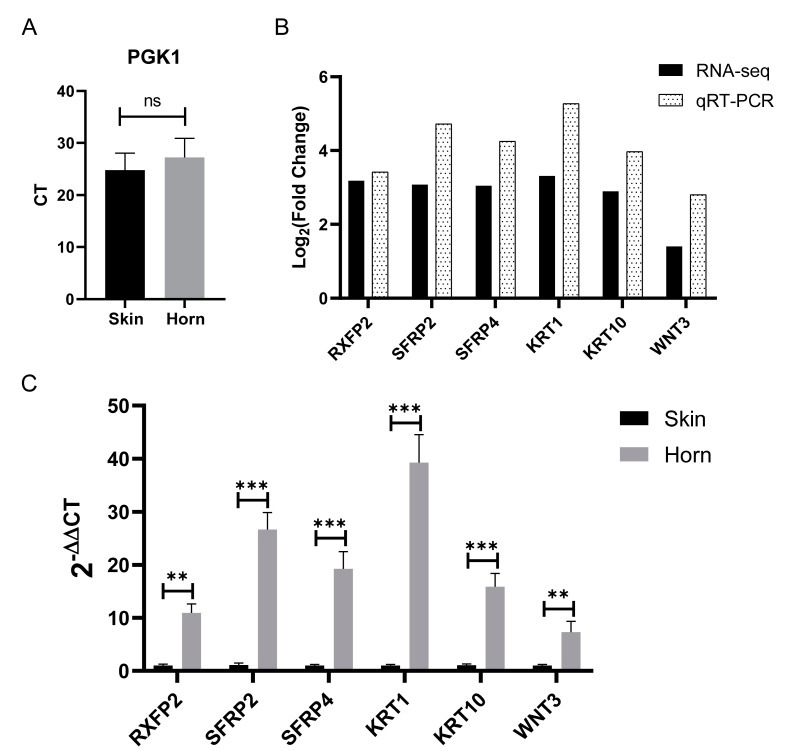
The verification of DEGs profile. (**A**) CT values of *PGK1* in horn buds and skin tissues. (**B**) Comparison of the fold changes between RNA-seq and qRT-PCR. (**C**) Gene expression of horn buds and skin tissues by qRT-PCR. ** *p* < 0.01, *** *p* < 0.001.

Further, we selected *PGK1* as an internal control. The CT value of *PGK1* showed no difference between the horn bud and skin tissue groups (Figure 9A), indicating that this gene was stably expressed in the two groups. *RXFP2*, *SFRP2*, *SFRP4*, *KRT1*, *KRT10*, and *WNT3* were selected as the six DEGs to be analyzed by qRT-PCR in order to determine their expression levels, which were to be used to validate the RNA-seq data. The results showed that the expression of these genes was significantly different between the two groups (Figure 9C). The gene expression patterns were consistent with the RNA-seq data (Figure 9B), suggesting that the transcriptome sequencing results are trustworthy.

## 4. Discussion

The embryonic period is crucial for the differentiation and formation of sheep horn [19]. However, little is known about horn development in sheep during this period. In this study, the histological results showed that the horn buds were significantly different from the forehead skin, having a thicker epidermis and more layers of epithelial cells, both of which findings have been previously reported [19,22]. However, the vacuolated keratinocytes previously found reported in the horn buds of cow fetuses were not observed in the present study [22]. Meanwhile, in a previous study of Merino sheep, the vacuolated keratinocytes were not found in the development of horn buds from 75 days of gestation to adulthood [19]. Therefore, it is necessary to collect earlier horn bud tissues for further analysis and verification.

Further, RNA-seq was performed in this study to investigate the gene expression profile of sheep horn buds during the embryonic stage. We found only 68 differentially expressed genes in the horn buds and forehead skin tissues, suggesting that horn development may be regulated by a few key genes during embryonic development. Further, our study identified four important candidate genes for ruminant horn formation in sheep fetal horn buds, namely *RXFP2*, *FOXL2, TNN*, and *ACAN*. The *RXFP2* gene was the most significant DEG identified (*p*-value = 7.42 × 10^−14^), and is a crucial candidate gene for the polled trait in sheep [8,12,13,29]. It is also related to horn size, length, and shape in sheep [5,7,8,9,16], indicating that it plays a vital role in horn development during the embryonic period. The *FOXL2* gene is involved in horn bud differentiation [36], and is associated with horn growth in goats [34]. Previous proteomic analysis has suggested that TNN is associated with the formation of horn deformity in sheep [17]. In addition, *TNN* is mainly associated with chondrogenesis during antler regeneration and acts as an important marker gene in antler blastema progenitor cells [37]. *ACAN* is a chondrogenic gene involved in cartilage development in antlers [37]. In this study, we also identified the *ACAN* gene, suggesting that this gene may also be involved in cartilage development in sheep horns. These results suggest that various previously identified horn-related genes, including *RXFP2*, *FOXL2*, *TNN*, and *ACAN*, play an important role in sheep horn development at the transcriptome level.

Additionally, our study provides some possible candidate genes for horn development, including the *KRT10*, *KRT1, FMOD*, free fatty acid receptor 4 (*FFAR4*), serine protease 22 (*PRSS22*), signal peptide, CUB domain, and EGF-like domain containing 2 (*SCUBE2*), extracellular matrix protein 1 (*ECM1*), and keratin 24 (*KRT24)* genes. The genes *KRT10* and *KRT1* are expressed in keratinocytes during early differentiation [38], and their mutations cause epidermolytic hyperkeratosis [39,40,41,42]. In this study, *KRT1* (FPKM = 3399) and *KRT10* (FPKM = 2037) were found to be highly expressed in the horn buds, implying that *KRT1* and *KRT10* may regulate the keratinization of horn buds. The *FMOD* gene not only promotes angiogenesis [43,44,45,46] but also regulates skin wound repair by transforming growth factor-β (TGF-β) [46,47,48]. This gene may regulate the development of blood vessels in horn buds and provide adequate nutrients for horn bud development. The genes *FFAR4*, *PRSS22*, and *SCUBE2* are associated with the epithelial–mesenchymal transition [49,50,51,52]. *ECM1* controls the differentiation of keratinocytes within the epidermis [53]. The *KRT24* gene has been suggested as an epidermal keratinocyte differentiation marker. [54]. These candidate genes may each play a role in horn development, including in epidermal keratinocyte differentiation and epithelial–mesenchymal transition; therefore, their functions need to be further validated.

According to our KEGG results, the DEGs identified were mainly enriched in the Wnt signaling pathway, which has been shown to play vital roles in epidermal development, stem cell self-renewal, and wound healing [55,56,57,58,59,60,61]. The horns of ruminants have a common cellular origin from neural crest cells [3] and the Wnt signaling pathway is essential for regulating the fate, migration, and proliferation of cranial neural crest cells [62,63,64,65]. In addition, antler regeneration may be influenced by the Wnt signaling pathway [37]. This suggests that the Wnt signaling pathway is important in the development and formation of horns. Interestingly, in this study, the hub genes *SFRP2*, *SFRP4*, *WNT3*, and *WNT7B* were found in the Wnt signaling pathway. *SFRP2* affects hair follicle and skin development through the Wnt signaling pathway, as well as in the catagen of hair follicles, where *SFRP2* inhibits keratinocyte proliferation [66,67]. Further, *SFRP2* is important for stem cell maintenance during antler regeneration [37]. *SFRP4* promotes epidermal differentiation and affects the function of osteoblasts and osteoclasts, promoting bone development and remodeling [68,69,70,71]. *WNT7B* is related to the epithelial–mesenchymal transition [72], and *WNT3* is associated with hair regeneration [73] and hair follicle development [74]. These results illustrate the potential role of these genes in the development of horn buds in sheep fetuses.

## 5. Conclusions

In our study, 68 DEGs were identified in the horn buds of sheep fetuses, including 32 horn-related genes identified in previous studies, such as *RXFP2*, *FOXL2*, *SFRP4*, *SFRP2*, *KRT1*, *KRT10*, *WNT7B*, and *WNT3*. *RXFP2* was found to be differentially up-regulated in the horn buds and recorded the highest significance. Furthermore, the DEGs were mainly enriched with regard to the growth, development, and cell differentiation terms. Additionally, KEGG enrichment analysis revealed that the DEGs were mainly involved in the Wnt signaling pathway. Therefore, the results indicated that the Wnt signaling pathway may be involved in horn development. This study provides new possible marker genes for horn development and promotes our understanding of horn formation.

## Figures and Tables

**Table 1:** Transcriptome data statistics.

Samples	Raw Reads	Clean Reads	Q30(%)	Mapping Rate
S1	42,148,762	40,337,970	91.73; 90.53	95.17%
S2	36,713,688	35,483,446	91.87; 90.82	95.53%
S3	42,799,612	41,443,883	92.17; 91.07	95.41%
H1	48,177,097	46,171,913	92.09; 90.90	93.49%
H2	38,016,087	36,458,805	91.17; 87.15	94.13%
H3	39,607,975	38,257,622	93.77;92.89	96.10%

## Data Availability

All clean data generated in this study were submitted to the National Center for Biotechnology Information Sequence Read Archive (NCBI SRA) database. The BioProject number is PRJNA947734.

## Supplementary Materials

The following supporting information can be downloaded at: https://www.mdpi.com/2079-7737/12/4/591/s1, Figure S1: KEGG pathway map for Wnt signaling pathway of DEGs; Table S1: Primer pairs of DEGs used for qRT-PCR validation; Table S2: The DEGs in horn bud and forehead skin tissues of fetal sheep; Table S3: Gene sets of horn-related genes and proteins from previous studies; Table S4: GO enrichment analysis of DEGs; Table S5: Top 5 hub genes of the protein–protein interaction network.
