# Correction: Luan et al. Identification of Critical Genes for Ovine Horn Development Based on Transcriptome during the Embryonic Period. *Biology* 2023, *12*, 591

**DOI:** 10.3390/biology12070915

**Published:** 2023-06-26

**Authors:** Yuanyuan Luan, Shangjie Wu, Mingkun Wang, Yabin Pu, Qianjun Zhao, Yuehui Ma, Lin Jiang, Xiaohong He

**Affiliations:** 1 Institute of Animal Sciences, Chinese Academy of Agricultural Sciences (CAAS), Beijing 100193, China; 82101205302@caas.cn (Y.L.);; 2 Key Laboratory of Livestock and Poultry Resources Evaluation and Utilization, Institute of Animal Sciences, Chinese Academy of Agricultural Sciences (CAAS), Beijing 100193, China

In the original publication [1], there were mistakes in the order of the references, which were as follows:

Lately, comparative transcriptome analysis has demonstrated that bovine horns and cervid antlers most likely share the same cellular origin, namely from neural crest stem cells [16].
Previous studies have demonstrated that sheep horn formation is initiated during the embryonic period [3,17].
RNA sequencing (RNA-seq) is a high-throughput technology that provides a comprehensive view of the entire transcriptome, which contributes to a better understanding of embryonic development and the role of genes [18,19].
The shape of sheep horn buds is similar to that of cattle horn buds [20].
The featureCounts program in Subread (v2.0.3) was used to calculate the gene expression of horn buds and skin tissues [21].
The package DESeq2 v1.36.0 of R software [22] was used to examine DEGs in horn buds and skin tissues.
This was carried out on the g: Profiler (https://biit.cs.ut.ee/gprofiler/gost (accessed on 15 September 2022)) and KEGG Mapper [23,24] (https://www.genome.jp/kegg/mapper/color.html (accessed on 15 September 2022)) tools.
The DEGs’ expression was compared to PGK1, an internal control [25], utilizing Livak and Schmittgen’s 2^−ΔΔCT^ technique to derive quantification cycle values [26].
Meanwhile, we integrated the gene sets (Table S3) and compared them with the DEGs identified in this study; these sets included the horn-related genes and proteins of sheep, goats, and cattle horns identified in previous studies [1,3,10,12–15,27–34].
The embryonic period is crucial for the differentiation and formation of sheep horn [17].
In this study, the histological results showed that the horn buds were significantly different from the forehead skin, having a thicker epidermis and more layers of epithelial cells, both of which findings have been previously reported [17,20]. However, the vacuolated keratinocytes previously found reported in the horn buds of cow fetuses were not observed in the present study [20]. Meanwhile, in a previous study of Merino sheep, the vacuolated keratinocytes were not found in the development of horn buds from 75 days of gestation to adulthood [17].
It is also related to horn size, length, and shape in sheep [5,7–9,35], indicating that it plays a vital role in horn development during the embryonic period. The *FOXL2* gene is involved in horn bud differentiation [36], and is associated with horn growth in goats [33]. Previous proteomic analysis has suggested that TNN is associated with the formation of horn deformity in sheep [27].
Venn diagram of DEGs and horn-related genes. A: DEGs; B: Horn-associated genes in transcriptome studies by Wang, Y. et al., Pannetier, M. et al., and Boulanger, L. et al.; C: Horn-associated genes in proteome studies by He, X. et al.; D: Horn-associated genes in genome studies by Li, Y. et al., Montgomery, G.W. et al., Kijas, J.W. et al., Wiedemar, N. et al., Luhken, G. et al., He, X. et al., Ren, X. et al., Pailhoux, E. et al., and Medugorac, I. et al.
23. Kanehisa, M.; Sato, Y.; Kawashima, M. KEGG mapping tools for uncovering hidden features in biological data. *Protein Sci.* **2022**, *31*, 47–53. https://doi.org/10.1002/pro.4172.
24. Kanehisa, M.; Furumichi, M.; Sato, Y.; Kawashima, M.; Ishiguro-Watanabe, M. KEGG for taxonomy-based analysis of pathways and genomes. *Nucleic Acids Res.* **2023**, *51*, D587–D592. https://doi.org/10.1093/nar/gkac963.
25. Liao, Y.; Smyth, G.K.; Shi, W. featureCounts: An efficient general purpose program for assigning sequence reads to genomic features. *Bioinformatics* **2014**, *30*, 923–930. https://doi.org/10.1093/bioinformatics/btt656.
26. Love, M.I.; Huber, W.; Anders, S. Moderated estimation of fold change and dispersion for RNA-seq data with DESeq2. *Genome Biol.* **2014**, *15*, 1–21. https://doi.org/10.1186/s13059-014-0550-8.

The corrected order of the references is as below:

Lately, comparative transcriptome analysis has demonstrated that bovine horns and cervid antlers most likely share the same cellular origin, namely from neural crest stem cells [18].
Previous studies have demonstrated that sheep horn formation is initiated during the embryonic period [3,19].
RNA sequencing (RNA-seq) is a high-throughput technology that provides a comprehensive view of the entire transcriptome, which contributes to a better understanding of embryonic development and the role of genes [20,21].
The shape of sheep horn buds is similar to that of cattle horn buds [22].
The featureCounts program in Subread (v2.0.3) was used to calculate the gene expression of horn buds and skin tissues [23].
The package DESeq2 v1.36.0 of R software [24] was used to examine DEGs in horn buds and skin tissues.
This was carried out on the g: Profiler (https://biit.cs.ut.ee/gprofiler/gost (accessed on 15 September 2022)) and KEGG Mapper [25,26] (https://www.genome.jp/kegg/mapper/color.html (accessed on 15 September 2022)) tools.
The DEGs’ expression was compared to PGK1, an internal control [27], utilizing Livak and Schmittgen’s 2^−ΔΔCT^ technique to derive quantification cycle values [28].
Meanwhile, we integrated the gene sets (Table S3) and compared them with the DEGs identified in this study; these sets included the horn-related genes and proteins of sheep, goats, and cattle horns identified in previous studies [1,3,10,12–15,17,29–35].
The embryonic period is crucial for the differentiation and formation of sheep horn [19].
In this study, the histological results showed that the horn buds were significantly different from the forehead skin, having a thicker epidermis and more layers of epithelial cells, both of which findings have been previously reported [19,22]. However, the vacuolated keratinocytes previously found reported in the horn buds of cow fetuses were not observed in the present study [22]. Meanwhile, in a previous study of Merino sheep, the vacuolated keratinocytes were not found in the development of horn buds from 75 days of gestation to adulthood [19].
It is also related to horn size, length, and shape in sheep [5,7–9,16], indicating that it plays a vital role in horn development during the embryonic period. The *FOXL2* gene is involved in horn bud differentiation [36], and is associated with horn growth in goats [34]. Previous proteomic analysis has suggested that TNN is associated with the formation of horn deformity in sheep [17].
Venn diagram of DEGs and horn-related genes. A: DEGs; B: Horn-associated genes in transcriptome studies by Wang, Y. et al. [3], Pannetier, M. et al. [33], and Boulanger, L. et al. [34]; C: Horn-associated genes in proteome studies by He, X. et al. [17]; D: Horn-associated genes in genome studies by Li, X. et al. [1], Montgomery, G.W. et al. [10], Kijas, J.W. et al. [12,31], Wiedemar, N. et al. [13], Lühken, G. et al. [14], He, X. et al. [15], Wang, X. et al. [29], Ren, X. et al. [30], Pailhoux, E. et al. [32], and Medugorac, I. et al. [35].
23. Liao, Y.; Smyth, G.K.; Shi, W. featureCounts: An efficient general purpose program for assigning sequence reads to genomic features. *Bioinformatics* **2014**, *30*, 923–930. https://doi.org/10.1093/bioinformatics/btt656.
24. Love, M.I.; Huber, W.; Anders, S. Moderated estimation of fold change and dispersion for RNA-seq data with DESeq2. *Genome Biol.* **2014**, *15*, 1–21. https://doi.org/10.1186/s13059-014-0550-8.
25. Kanehisa, M.; Sato, Y.; Kawashima, M. KEGG mapping tools for uncovering hidden features in biological data. *Protein Sci.* **2022**, *31*, 47–53. https://doi.org/10.1002/pro.4172.
26. Kanehisa, M.; Furumichi, M.; Sato, Y.; Kawashima, M.; Ishiguro-Watanabe, M. KEGG for taxonomy-based analysis of pathways and genomes. *Nucleic Acids Res.* **2023**, *51*, D587–D592. https://doi.org/10.1093/nar/gkac963.

The authors state that the scientific conclusions are unaffected. This correction was approved by the Academic Editor. The original publication has also been updated.
