# Supplementary material for: Identification of Critical Genes for Ovine Horn Development Based on Transcriptome during the Embryonic Period
Source: Biology (Basel). 2023 Apr 13;12(4):591. doi: 10.3390/biology12040591 (PMC10136283; doi:10.3390/biology12040591)
Supplement: Supplementary file 1 [file biology-12-00591-s001.zip › Supplementary Figure.pdf]

Supplementary Figure

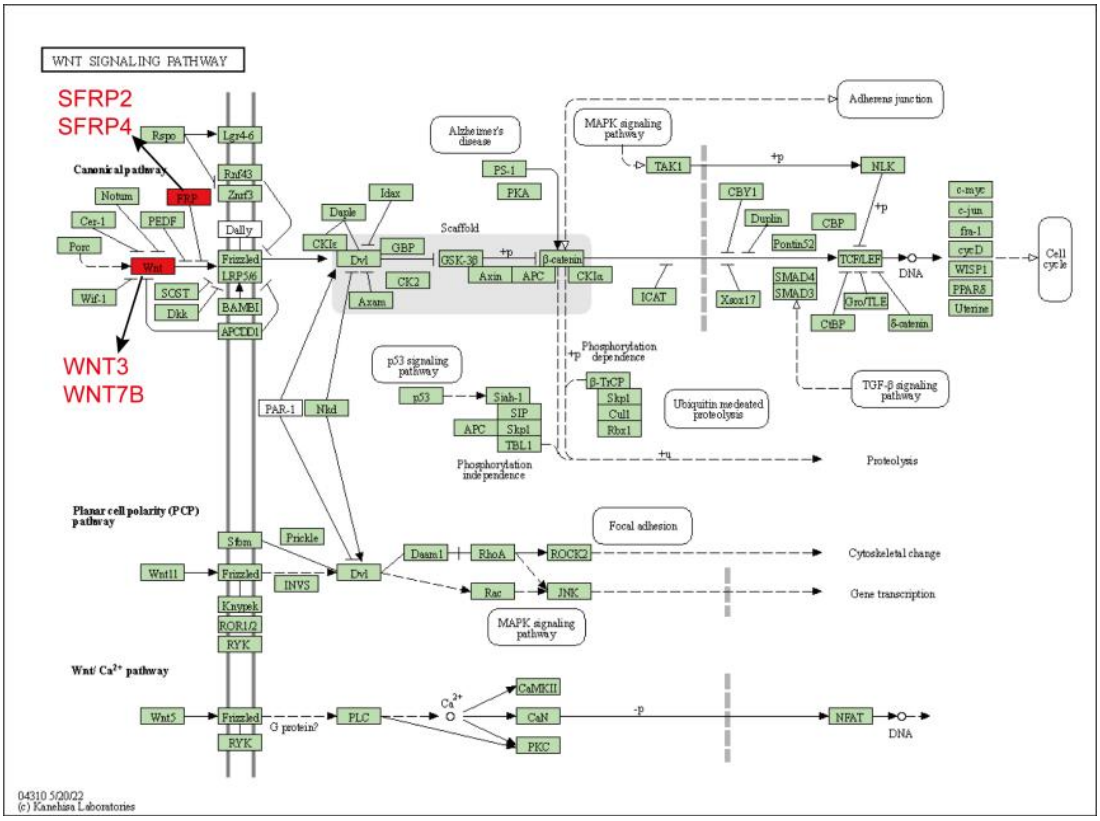

Supplementary Figure S1. KEGG pathway map for Wnt signaling pathway of DEGs.
